# Naturally acquired antibodies from Beninese infants promote *Plasmodium falciparum* merozoite-phagocytosis by human blood leukocytes: implications for control of asymptomatic malaria infections

**DOI:** 10.1186/s12936-022-04361-w

**Published:** 2022-11-29

**Authors:** Abdou Khadre Dit Jadir Fall, Ikhlaq Hussain Kana, Célia Dechavanne, Asier Garcia-Senosiain, Evelyne Guitard, Jacqueline Milet, Achille Massougbodji, André Garcia, Jean-Michel Dugoujon, Florence Migot-Nabias, Michael Theisen, David Courtin

**Affiliations:** 1 Université Paris Cité, IRD, MERIT, 75006 Paris, France; 2 Department for Congenital Disorders, Statens Serum Institut, Copenhagen, Denmark; 3 Centre for Medical Parasitology, Department of International Health, Immunology and Microbiology, University of Copenhagen, Copenhagen, Denmark; 4 Laboratoire d’Anthropologie Moléculaire et Imagerie de Synthèse, UMR 5288, Université Paul Sabatier Toulouse III, 37 Allées Jules-Guesde, 31073 Toulouse, France; 5 Centre d’Etude et de Recherche sur les Pathologies Associées à la Grossesse et à l’Enfance, Cotonou, Bénin

**Keywords:** IgG, Gm allotypes, Opsonic phagocytosis, Control of asymptomatic malaria infection, Benin

## Abstract

**Background:**

Immunoglobulin G (IgG) antibodies are thought to play important roles in the protection against *Plasmodium falciparum* (*P. falciparum*) malaria. A longitudinal cohort study performed in the Southern part of Benin, identified a group of infants who were able to control asymptomatic malaria infections (CAIG).

**Methods:**

IgG antibodies against distinct merozoite antigens were quantified in plasma from Beninese infants. Functionality of these antibodies was assessed by the merozoite-phagocytosis assay using THP-1 cells and primary neutrophils as effector cells. Gm allotypes were determined by a serological method of haemagglutination inhibition.

**Results:**

Purified IgG from infants in CAIG promoted higher levels of merozoite-phagocytosis than did IgG from children who were unable to control asymptomatic infections (Ologit multivariate regression model, Coef. = 0.06, 95% CI 0.02;0.10, P = 0.002). High level of merozoite-phagocytosis activity was significantly associated with high levels of IgG against AMA1 (Coef. = 1.76, 95% CI 0.39;3.14, P = 0.012) and GLURP-R2 (Coef. = 12.24, 95% CI 1.35;23.12, P = 0.028). Moreover, infants of the G3m5,6,10,11,13,14,24 phenotype showed higher merozoite-phagocytosis activity (Generalized linear model multivariate regression, Coef. = 7.46, 95% CI 0.31;14.61, P = 0.041) than those presenting other G3m phenotypes.

**Conclusion:**

The results of the present study confirm the importance of antibodies to merozoite surface antigens in the control of asymptomatic malaria infection in Beninese infants. The study also demonstrated that G3m phenotypes impact the functional activity of IgG. This last point could have a considerable impact in the research of candidate vaccines against malaria parasites or other pathogens.

**Supplementary Information:**

The online version contains supplementary material available at 10.1186/s12936-022-04361-w.

## Background

Malaria is caused by parasites of the *Plasmodium* genus. Of these, *Plasmodium falciparum* causes the most severe forms of malaria and is the primary cause of malaria related mortality and morbidity in children under 5 years of age in sub-Saharan Africa [1]. Immune epidemiological studies have shown that children living in malaria endemic areas may develop naturally acquired immunity against *P.* *falciparum* malaria after repeated infections [2, 3]. However, this immunity is non-sterile and can be lost if immune individuals are not exposed to malaria [4, 5].

To study the development of immunity against clinical malaria during infancy, samples and data were collected from children residing in Tori Bossito in Benin from birth to 18 months of age. The analyses of clinical and parasitological data obtained during the follow-up allowed us to identify a group of infants who were able to clear asymptomatic infections or at least to reduce the *P. falciparum* parasitaemia below the detection threshold of thick blood smear (CAIG) as determined by monthly thick blood smears (TBS) [6]. These children experienced a single asymptomatic infection and were TBS negative at the subsequent monthly visit(s). Importantly, they did not present any symptoms of clinical malaria during the remaining period of follow-up. Infants in CAIG were able to control not only parasite multiplication, but also disease symptoms. Moreover, these children showed higher levels of specific antibodies (Ab) to asexual blood-stage antigens (AMA1, MSP1, MSP2-FC27, MSP3 and GLURP-R2) than children who were unable to control malaria asymptomatic infection [6].

It is generally accepted that antibodies of the cytophilic subclasses (IgG1 and IgG3) may be particularly important in protection against clinical malaria [7–12]. Accordingly, high levels of IgG against merozoite-associated antigens (AMA1, MSP1, MSP2, MSP3 and GLURP) were associated with a reduced risk of *P. falciparum* malaria infections while other correlated these IgG to malaria risk or exposition [6, 13–15]. Such antibodies may act either directly by inhibiting invasion of red blood cells or indirectly by controlling parasite multiplication through collaboration with immune cells expressing Fc gamma receptors (FcgR) [16]. In FcgR mediated activities (phagocytosis, ADCI and ADCC), the malarial antibody first binds to its antigen on the target cells, and then its Fc portion binds to the FcgR on the effector cell. Significant correlations between opsonic phagocytosis (OP) of merozoites and immunity against clinical malaria were previously reported [9, 17, 18]. Of the various effector cells, neutrophils appear to be particularly important in this respect [19]. Neutrophils are generally the first circulating cells and have been shown to phagocytose *P. falciparum*-infected red blood cells (iRBC) in vivo, free circulating merozoites and occasionally trophozoites [20–23].

Gm allotypes are allelic variations in the CH1, CH2 and CH3 IgG heavy chain, that induce alterations in the amino acid sequences which may affect their binding to FcgRs [24]. Gm allotypes are inherited in fixed combinations called haplotypes and vary qualitatively and quantitatively according to human population groups [25]. The most polymorphic G3m allotypes are distinguished by variations on nine amino acids (http://www.imgt.org/). Gm allotypes have been shown as implicated in malaria susceptibility [24, 26, 27], but their role in OP activity needs to be investigated.

Here, samples and data were used from a well-established birth-cohort from Benin, to investigate the functional activity of serum IgG antibodies. Infants were typed with respect to their Gm allotypes and specific antibodies (IgG, IgG1 and IgG3) against a panel of merozoite-antigens were quantified by ELISA. The functional activity of IgG was investigated in the merozoite-phagocytosis assay. IgG levels and specific G3m phenotypes were strong predictors of the merozoite-phagocytosis activity by monocytes and neutrophils, respectively.

## Methods

### Study design

The study was conducted in the district of Tori Bossito in southwest Benin between July 2007 and January 2010, where 567 children were followed-up from birth to 18 months of age. This follow-up consisted in an epidemiological, parasitological and immunological follow-up detailed elsewhere [26–28]. Health workers recruited by the program visited the infants at home every week in order to check their health status. In case of fever (axillary temperature  > 37.5 °C), a rapid diagnostic test (RDT) and/or thick blood smear examination (TBS) were performed. In case of symptomatic malaria (fever and positive RDT/TBS), an anti-malarial treatment (artemether/lumefantrine) was administered according to the national guidelines that were applied at the time of the study.

TBS were also performed monthly to detect asymptomatic malaria infections. In addition, mothers were invited to bring their infants to the health centre, at any time, in case of fever (suspected by the mother) or clinical signs, whether or not related to malaria, and the same protocol was applied. Venous blood samples were collected quarterly for hematological and immunological measurements. Venous blood was centrifuged for plasma isolation and genomic DNA extraction. IgG1 and IgG3 responses against *P. falciparum* vaccine antigen candidates AMA1, MSP1, MSP3, MSP2-3D7, MSP2-FC27, GLURP-R0 and GLURP-R2 were quantified at 6, 9, 12, 15 and 18 months by ELISA [6]. IgG purification was performed for the 106 plasma samples with sufficient volumes at 15th or 18th month time-points. Among them, 27 samples belonged to the group of infants able to control asymptomatic malaria infections over time (CAIG).

The control of asymptomatic malaria infection was defined as follow: the clearance of blood *P. falciparum* parasites or at least maintain *P. falciparum* at very low parasitaemia, below the detection threshold of thick blood smear [6]. CAIG group was compared with two other groups: a group of 53 children infected by *P. falciparum* but not able to control asymptomatic malaria infection (IG group) and a group of 23 children for which *P. falciparum* has not been detected by TBS examination during the survey (N*Pf*DG group). These two groups were composed of all other infants for whom sufficient plasma volume was available.

### IgG purification and IgG controls

Ab SpinTrap^™^ columns containing protein G coupled to Sepharose were used to purify IgG from 60 μl of plasma diluted 1/10 in phosphate buffered saline (PBS), as per manufacturer's instructions (Sigma-Aldrich). IgG concentration was measured using a NanoDrop®ND-1000 spectrophotometer, and samples were stored at −80 °C. Purified IgG samples were diluted in PBS to obtain a final concentration of 50 ng/mL before performing OP assays. Pooled IgG from malaria-exposed hyperimmune Liberian adults or malaria-non immune Danish blood donors served as internal controls [29].

### *Plasmodium falciparum* culture and preparation of merozoites

*P. falciparum* line NF54 was cultured and merozoite isolation was performed as described previously [30]. Briefly, parasites were maintained in O + human erythrocytes at 3–4% haematocrit in parasite growth medium (RPMI 1640 Gibco™ supplemented with 25 mM HEPES, 0.5% AlbuMAX, 4 mM l-glutamine, 0.02 g/l hypoxanthine, and 25 μg/ml gentamicin) at 37 °C in a humidified 5% CO_2_, 2% O_2_ and 93% N_2_ atmosphere. The parasitaemia was monitored by examination of Giemsa-stained thin blood smears, and parasite cultures were synchronized with 5% sorbitol treatment for 10 min. Synchronized trophozoite-stage parasites were harvested using a magnetic separation column (Vario MACS) and subsequently treated with 10 μM of epoxysuccinyl-l-leucylamido (4-guanidino) butane (E64) for 6–8 h to allow schizonts to mature without rupture. Segmented schizonts were centrifuged, resuspended in 4–6 ml RPMI 1640 medium, and then filtered through a 1.2 μm/32 mm syringe filter to obtain merozoites. The filtrate was passed over an LS MACS Column twice to remove the haemozoin. Free merozoites were stained with 10 μg/ml of ethidium bromide (EtBr) for 30 min before used in OP assay.

### Human neutrophil purification and culture of THP-1 cells

Primary neutrophils were purified from freshly drawn blood from a healthy Danish donor as described previously [17]. Briefly, blood was layered over a Percoll solution (1.077 g/ml) and centrifuged at 800 g for 25 min. The granulocyte-containing band was transferred to ten volumes of RBC lysis solution (155 mM NH_4_Cl, 10 mM KHCO_3_ and 0.1 mM EDTA) and incubated for 10 min.

The cells were centrifuged and further purification of neutrophils was carried out with the EasySep Human Neutrophil Isolation Kit (StemCell Technologies) according to the manufacturer’s protocol. For OP assay, neutrophils were resuspended in cell medium (RPMI 1640, 10% fetal bovine serum (FBS), 4 nM l-glutamine and 25 μg/ml gentamicin) and distributed in 96-well, U-bottom plates (1 × 10^5^ cells/150 µl/well). The human monocytic cell line THP-1 was maintained as described previously [31]. Likewise, neutrophils, viable THP-1 cells were distributed in 96-well, U-bottom plates (1 × 10^5^ cells/150 µl/well).

### Opsonic phagocytosis assay measured by flow cytometry

The OP assay was performed as described in detail [16]. Briefly, freshly isolated EtBr-stained merozoites were opsonized with 0.05 mg/ml IgG (controls and test samples) for 20 min. Aliquots of 50 μl of opsonized merozoites were co-incubated with pre-seeded primary neutrophils or THP-1 cells. The plates were incubated for 35 min at 37 °C in a 5% CO_2_ humidified incubator. To stop phagocytosis, plates were centrifuged for 5 min at 400 g at 4 °C, followed by 2 washes with cold fluorescence-activated cell sorting (FACS) buffer (PBS + 0.5% BSA + 2 mM EDTA). Then, cells were fixed with 200 μl of FACS fixative (FACS buffer + 2% paraformaldehyde) and kept at 4 °C until analysed in a CytoFLEX S flow cytometer (Beckman Coulter). Viable neutrophils or THP-1 cells were gated by forward scatter and side scatter properties, and EtBr-positive events were enumerated using primary neutrophils or THP-1 cells alone [16]. The percentage of primary neutrophils or THP-1 cells containing EtBr-stained merozoites determined the level of phagocytosis, which was expressed as the phagocytosis index (PI). Data analyses were performed with Kaluza Analysis Software (version 2.1).

### IgG Gm phenotypes

The determination of Gm allotypes was performed before our study and included in our statistical analyses. Detailed description on the determination of IgG Gm allotypes of the Tori Bossito cohort is available elsewhere [26]. Briefly, the Gm allotype determination was performed for infants for whom a sufficient quantity of plasma (200 µl) was available at 15 months of age. The Gm allotype diversity of infants was confirmed by means of its consistency (inheritance) with this of both biological parents. G1m [1–3, 17] and G3m [5, 6, 10, 11, 13–16, 21, 24, 28] allotypes were determined in plasma samples by a standard haemagglutination inhibition method which allowed to consider resulting Gm phenotypes [31]. Due to the constant presence of the G1m1,17 phenotype in the population group under study, only the G3m phenotype diversity was considered.

Considering G3m allotypes, no child presented G3m16 or G3m21, both located on the CH2 domain of the IgG heavy chain, and absent in populations from sub-Saharan Africa. Similarly, due to the uncertain location of the Gm28 allotype on either IgG1 or IgG3 sub-classes among sub-Saharan Africans [17], this allotype was discarded from the analysis. Therefore, the differentiation of infants was based on the remaining G3m allotypes combined into four G3m alleles mostly encountered in Africa, that are G3m5,10,11,13,14, G3m5,6,11,24, G3m5,6,10,11,14 and G3m10,11,13,15 [17]. The homozygous or heterozygous carriage of these alleles led to the ten-following possible G3m phenotypes: G3m5,10,11,13,14; G3m5,6,10,11,14; G3m5,6,10,11,13,14; G3m5,6,11,24; G3m5,6,10,11,13,14,24; G3m5,6,10,11,14,24; G3m10,11,13,15; G3m5,10,11,13,14,15; G3m5,6,10,11,13,14,15 and G3m5,6,10,11,13,15,24.

### IgG quantification

IgG quantification was performed before our study and included in the statistical analyses.

The Enzyme Linked Immunosorbent Assay (ELISA) was used to assess antibody levels against seven merozoite antigens. Details about the recombinant proteins and the procedure are described elsewhere [13, 29]. Briefly, recombinant proteins (0.1 μg/well) diluted in PBS were coated on MaxiSorp Nunc plates and blocked with 3% powdered-milk 0.1% PBS-Tween 20. For total IgG quantification, plasma samples were tested at 1:500 (for AMA1) and 1:100 (for MSP2-3D7, MSP2-FC27, MSP1, MSP3, GLUR-R0 and GLURP-R2). To quantify cytophilic IgG subclasses (IgG1 and IgG3), samples were used at 1:50 dilution against all the antigens. Horseradish peroxidase-conjugated anti-human IgG1 (NL16 clone) at 1:2000 dilution and anti-human IgG3 (ZG4 clone) at 1:5000 dilution (Skybio, France) were used as detection antibodies. The plates were washed thrice with PBS Tween-20 (0.1%) NaCl (0.5 M) after blocking, primary and detection antibodies. ADAMSEL software (Auditable Data Analysis and Management System for ELISA) was used to transform optical density (OD) values into antibody concentrations [32].

### Statistical analyses

The statistical analysis was carried out using the Stata software version 13. Comparisons of demographic and clinical characteristics were investigated between the CAIG, IG and N*Pf*DG groups using Chi-square test for categorical variables and Mann–Whitney *U*-test and Kruskal–Wallis test for quantitative variables. Kruskal–Wallis test was used to determine if the distribution of OP values was different between groups while the Chi-square test compared the proportion of infants with low/high OP (based on the median OP values) between groups.

Finally, the correlation between OP values, groups (CAIG, IG and N*Pf*DG), antibody levels against *P. falciparum* merozoite antigens and G3m phenotypes carriages were studied by multivariate regressions. Multivariate regressions studied the correlation between OP values, groups, antibody levels and G3m phenotypes using control variables. In this study, only control variables with P < 0.20 among demographic and clinical characteristics were specifically used. The correlation between OP values and groups (CAIG, IG and N*Pf*DG) was studied using a multivariate ordered logit regression model; N*Pf*DG, IG, and CAIG was coded 0, 1 and 2, respectively, according to OP values observation. If the coefficient and P value was significant and positive, the OP values increased with groups in the order 0, 1 and 2 otherwise the OP values did not increase with groups. The association between OP values, IgG levels and G3m phenotypes were studied using a generalized linear multivariate regression model where only control variables with P < 0.20 among demographic and clinical characteristics were specifically used. In these models, validity assumptions (normality, heteroscedasticity) have been checked and also the deviance and Pearson were shown as regards the model fitting.

### Ethics

The Faculté des Sciences de la Santé institutional review board and the IRD’s Consultative Ethics Committee approved the study protocol. All mothers in this study signed an informed consent before enrollment (which also included their infants) with the possibility to withdraw at any time.

## Results

### Characteristics of the study group

**Fig. 1:**
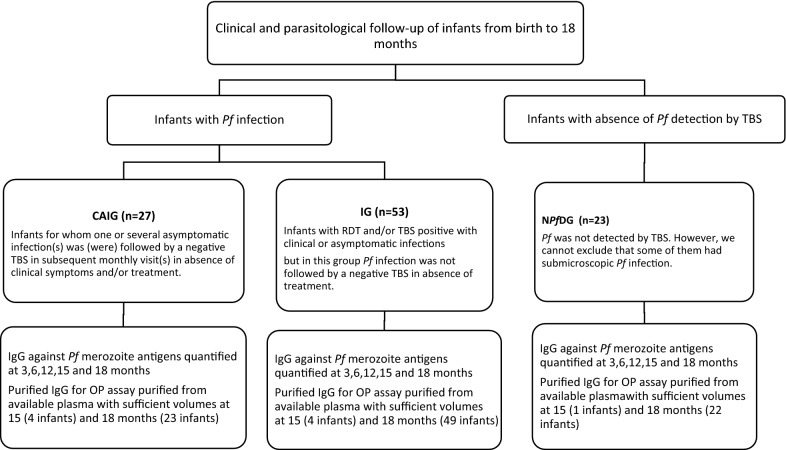
Group definitions

**Fig. 2:**
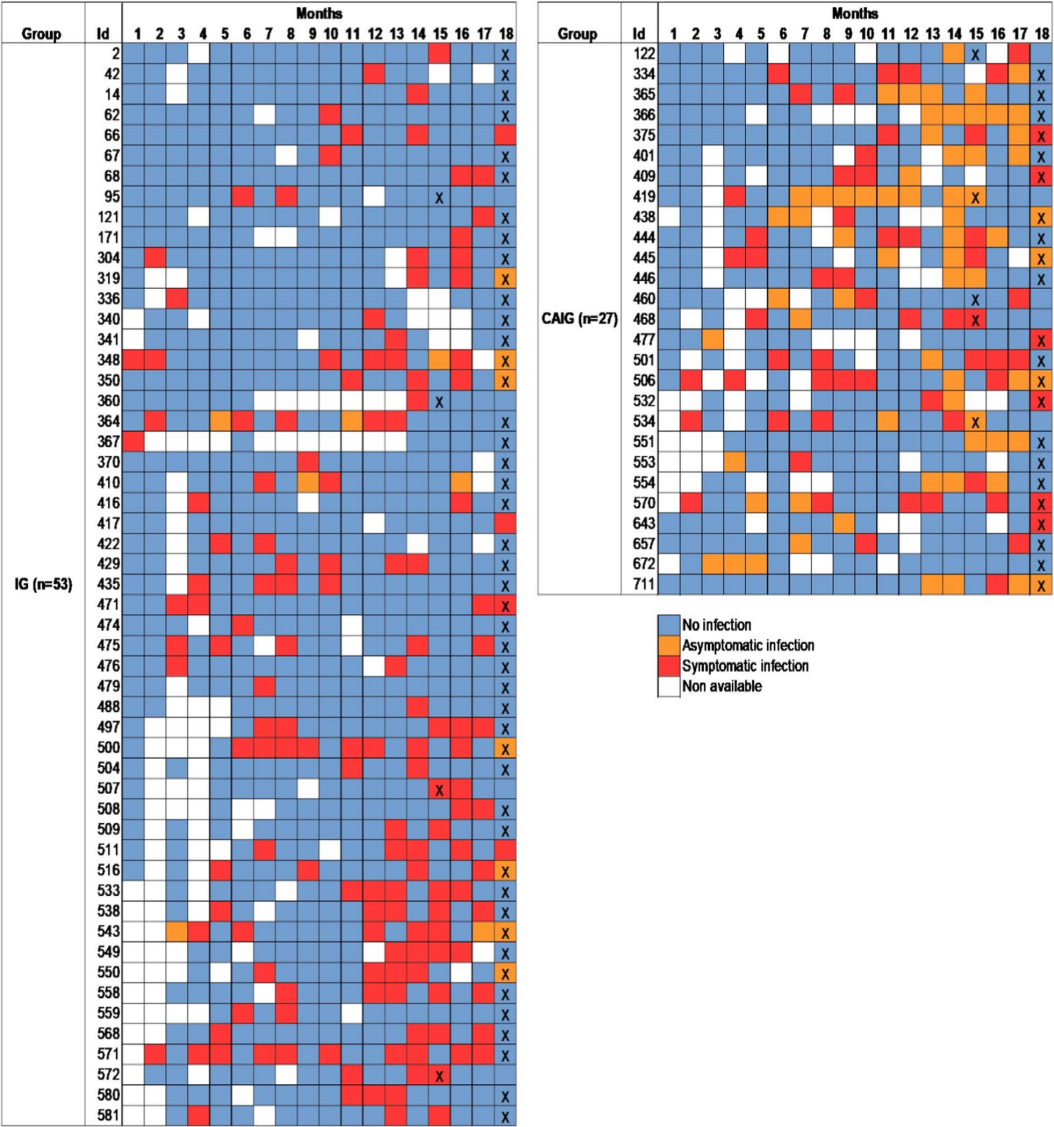
Monthly timeline of infections for children in IG and CAIG groups. For each child, squares represent 1 month of the follow-up (from birth to 18 months). The color of the squares indicates when asymptomatic (orange) and symptomatic (red) infections occurred. Some monthly TBS were not available because the child was not present at the scheduled visit or because the result of the TBS was not available. Cross indicates the time-point of plasma used for OP determination

A total of 567 children from Tori Bossito in southwest Benin where followed-up from birth to 18 months of age [28]. During the follow up period, 61% of children had at least one febrile malaria infection and 24% of children at least one asymptomatic infection. For this study, IgG samples and clinical data were available from 103 children (Fig. 1). Twenty-three children, remained parasite-negative by TBS throughout the study (N*Pf*DG) and 80 children tested parasite-positive at one or more of the monthly visits. Of the 80 parasite-positive children, 27 were able to clear asymptomatic malarial infections or to reduce *P. falciparum* parasitaemia under detection threshold of thick blood smear (CAIG) while 53 had *P. falciparum* infection with clinical symptoms (IG) (Fig. 1). The timeline of *P. falciparum* infections in each group was detailed in Fig. 2.

Infants from CAIG had more asymptomatic infections (P < 0.001) when compared to infants from IG while there was no difference regarding symptomatic infections between the two groups (P = 0.209, Table 1). Study participants belonged mostly to the Tori ethnic group with no differences in the distribution of ethnic groups between CAIG, IG and N*Pf*DG groups (P = 0.121).

**Table 1:** Characteristics of the study group

Characteristics of the study group	CAIG group (n = 27)	IG group (n = 53)	N*Pf*DG group (n = 23)	P-value
Symptomatic *Pf* infection (median Q1–Q3)	2 (0–4)	2 (1–3)		0.209^a^
Asymptomatic *Pf* infection (median Q1–Q3)	2.5 (1–4)	1 (0–1)		<** 0.001**^a^
Mothers				
Number of infants. (Median Q1–Q3)	3 (1–4)	3 (2–5)	4 (2–6)	0.386^C^
Maternal age (median Q1–Q3)	26 (22–30)	27.5 (23–30)	31 (29–35)	** 0.001**^C^
Number of antenatal visits (Median Q1–Q3)	3 (2–4)	3 (1–5)	5 (4–6)	**0.005**^C^
Number of Preventive malaria treatment (median Q1–Q3)	1 (0–1)	1 (1–1)	1 (1–1)	0.514^C^
Infants				
Birth weight (Median Q1–Q3)	2901 (2702.5–3212.5)	2957.5 (2637.5–3162.5)	3175 (2860–3315)	0.086^C^
Ethnic group (n. %):				0.121^b^
Aïzo	17 (68)	42 (79)	15 (65)	
Fon	5 (20)	7 (13)	2 (7)	
Others	3 (12)	3 (8)	6 (28)	
Bednet possession (Median Q1–Q3)	1 (0–1)	1 (0–1)	1 (1–1)	**0.049**^C^
HbS (n. %):				< **0.001**^b^
AA	14 (56)	38 (81)	20 (87)	
AS	10 (40)	9 (19)	3 (13)	
SS	1 (4)	0 (0)	0 (0)	
Bednet use for infants (Median Q1–Q3)				
Score ranging from 1 (rare use) to 4 (frequent use)	4 (3–4)	4 (3–4)	3 (3– 4)	0.429^C^

This table presents the groups of the study. CAIG is the group of infants able to control malaria asymptomatic infection, IG is the group of infants with symptomatic and/or asymptomatic infections but not able to control malaria asymptomatic infections and N*Pf*DG is the group of infants for which *Pf* has not been detected by TBS examination during the survey

^a^Statistical significance determined using Mann–Whitney *U*-test between IG and CAIG groups

^b^Statistical significance determined by χ2 analysis

^C^Statistical significance determined by Kruskal–Wallis test

In bold: significant P value at the 0.05 threshold

The distribution of bed net use and gender was similar between the three groups (P = 0.429). In contrast, the age of the mothers (P = 0.001) and the number of antenatal visits (P = 0.005) was highest in N*Pf*DG and the number of HbS carriers was highest in CAIG (P = 0.011) (Table 1).

### Merozoite-phagocytosis by different effector cells

Merozoite-phagocytosis assays are typically performed with primary cells or with the THP1 cell line [33–35]. To compare the activity of different effector cells, primary neutrophils and THP1 cells were used to assess the capacity of infants IgG to promote merozoite-phagocytosis.

**Fig. 3:**
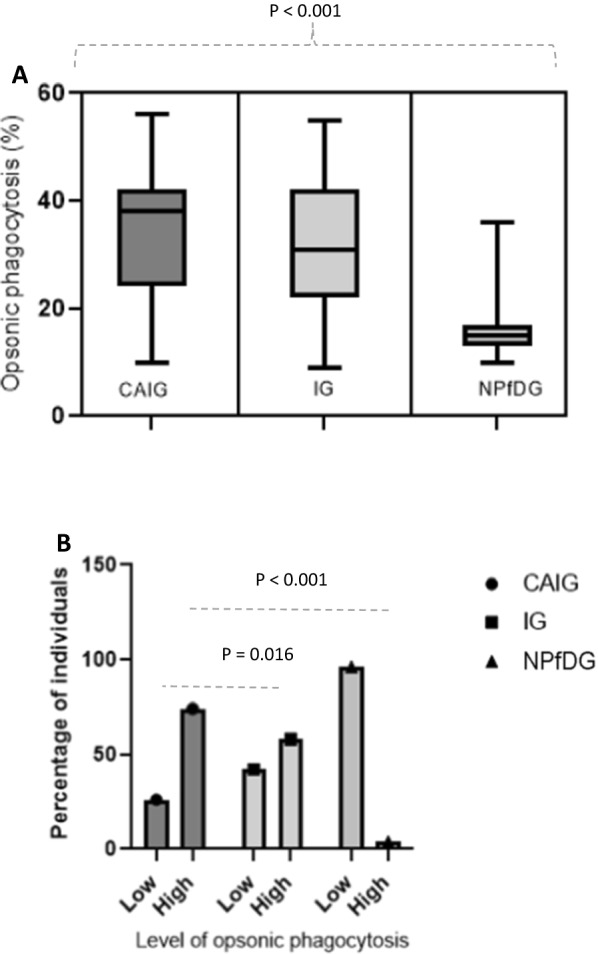
OP values in CAIG, IG and N*Pf*DG groups of children with THP-1 cells. **A** compares the distribution of OP with THP-1 cells between groups and **B** compares the percentage of low and high OP between groups. Kruskal–Wallis test was used to compare the distribution of OP between groups and Chi square test the proportion of high OP between CAIG and other groups

Freshly purified merozoites were stained with EtBr, opsonized with purified IgGs, and added to the respective effector cells. Merozoite-phagocytosis was assessed by FACS analysis and quantified as the proportion of cells with EtBr-stained merozoites. Using THP-1 as effector cells, there was a significant difference in merozoite-phagocytosis activities between groups (Fig. 3A, Kruskal–Wallis test, P < 0.001). Of the three groups, CAIG had the highest merozoite-phagocytosis activity (n = 20/27, 74%) compared to the IG (n = 31/53, 58%, Chi-square test, P = 0.016) and N*Pf*DG groups (n = 1/23, 4%, Chi-square test, P < 0.001), respectively (Fig. 3B). The Ologit multivariate regression model showed an increasing OP value in CAIG groups compared to other two groups Coef. = 0.06, 95% CI 0.02; 0.10, P = 0.002) (Table 2).

**Table 2:** Association between OP and the malaria status of the children according to their belonging to the CAIG, N*Pf*DG and IG groups

N*Pf*DG/IG/CAIG	Coef.	95% CI	P value	R^2^	Number of observations
OP THP-1 Model					
OP values	0.06	0.02; 0.10	**0.002**	0.22	94
OP Neutrophil Model					
OP Values	0.04	0.01; 0.07	**0.001**	0.23	94

This table presents the ordered logit model (Ologit) obtained through the control variables bednet use, sex, maternal age and and HbS. The dependent variable was the status N*Pf*DG / IG / CAIG coded, respectively by 0,1 and 2

In bold: significant P value < 0.05

**Fig. 4:**
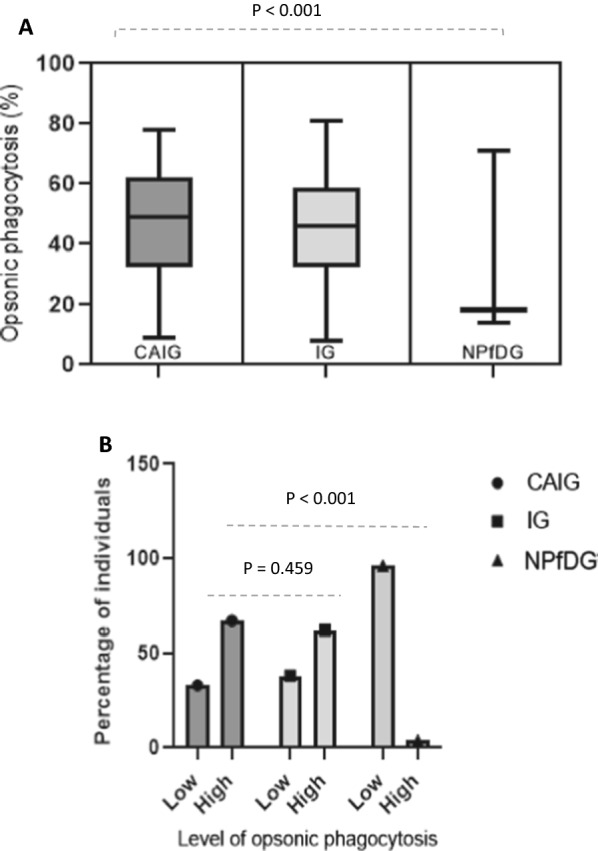
OP values in CAIG, IG and N*Pf*DG with primary neutrophils. **A** compares the distribution of OP with primary neutrophils cells between groups and **B** compares the percentage of low and high OP between groups. Kruskal–Wallis test was used to compare the distribution of OP between groups and Chi square test the proportion of high OP between CAIG and other groups

The same pattern was observed when using primary neutrophils as the effector cells: there was a significant difference in merozoite-phagocytosis activities between groups (Fig. 4A, Kruskal–Wallis test, P < 0.001), CAIG had highest level of high OP values compared to N*Pf*DG (Fig. 4B, Chi-square test, P < 0.001) and IG group even the difference did not reach statistical significance (Fig. 3B, 67% vs 62%, chi-square P = 0.459). Finally, the Ologit multivariate regression model showed as when using THP-1 as effector cells that OP values increased between groups (Coef. = 0.04, 95% CI.0.01; 0.07, P = 0.001) (Table 2).

### Merozoite-phagocytosis and specific IgG levels

In an attempt to the delignate potential antigens involved in merozoite-phagocytosis activity, levels of specific antibodies (IgG, IgG1, and IgG3) against AMA1, MSP2-3D7, MSP2-FC27, MSP1, MSP3, GLURP-R0, and GLURP-R2 were quantified by ELISA (Additional file 1: Table S1). First, the association between merozoite-phagocytosis by THP1 and levels of specific IgG was investigated (Tables 3–5). There was a positive correlation between merozoite-phagocytosis activity and levels of IgG against AMA1 (Coef. = 1.76, 95% CI 0.39; 3.14, P = 0.012) and GLURP-R2 (Coef. = 12.24, 95% CI 1.35;23.12, P = 0.028), respectively (Table 3).

**Table 3:** Association between OP and total IgG to merozoite *P. falciparum* antigens

Opsonic phagocytosis	Coef.	95% CI	P value	
Model 1: OP THP-1				
AMA1	1.76	0.39;3.14	**0.012**	Number of observations = 87
MSP1	0.6	−1.34;2.54	0.544	Adj R^2^ = 0.28
MSP3	16.56	−4.63;37.76	0.126	
MSP2-3D7	−3	−6.99;0.98	0.140	
MSP2-FC27	1.04	−3.47;5.54	0.652	
GLURP-R0	−20.80	−50.45;8.84	0.169	
GLURP-R2	12.24	1.35;23.12	**0.028**	
Model 2: OP Neutrophils				
AMA1	2.3	0.06;4.54	**0.044**	Number of observations = 87
MSP1	0.67	−2.49;3.84	0.677	Adj R^2^ = 0.21
MSP3	23.89	−8.71;34.45	0.176	
MSP2-3D7	−2.27	−6.27;1.99	0.493	
MSP2-FC27	1.60	−5.59;3.80	0.669	
GLURP-R0	17.65	−57.45;2.74	0.474	
GLURP-R2	5.09	1.67;23.62	0.574	

This table presents the generalized linear model obtained through the control variables bednet use, sex, and HbS. Total IgG to merozoite *P. falciparum* antigens were quantified at 18 months of age

In bold: significant P value at the 0.05 threshold

**Table 4:** Association between OP and IgG1 to merozoite *P. falciparum* antigens

Opsonic phagocytosis	Coef.	95% CI	P value	
Model 1: OP THP-1				
IgG1_AMA1				Number of observations = 87
High	9.38	2.38;16.39	**0.009**	Adj R^2^ = 0.30
Low (reference)				
IgG1_MSP1				
High	7.71	1.81;13.61	**0.010**	
Low (reference)				
IgG1_MSP3				
High	−1.19	−8.40;6.00	0.744	
Low (reference)				
IgG1_MSP2-3D7				
High	3.72	−3.48;10.93	0.311	
Low (reference)				
IgG1_MSP2-FC27				
High	5.54	−1.91;13	0.145	
Low (reference)				
IgG1_GLURP-R0				
High	0.06	−5.69;5.82	0.983	
Low (reference)				
IgG1_GLURP-R2				
High	−2.48	−9.61;4.64	0.494	
Low (reference)				
Model 2: OP Neutrophils				
IgG1_AMA1				Number of observations = 87
High	19.87	9.33;30.40	**0.0001**	Adj R^2^ = 0.27
Low (reference)				
IgG1_MSP1				
High	13.21	4.33;22.09	**0.004**	
Low (reference)				
IgG1_MSP3				
High	−5.88	−16.72;4.94	0.287	
Low (reference)				
IgG1_MSP2-3D7				
High	1.69	−9.15;12.53	0.760	
Low (reference)				
IgG1_MSP2-FC27				
High	5.15	−12.90;4.41	0.368	
Low (reference)				
IgG1_GLURP-R0				
High	−4.24	−13.39;8.06	0.367	
Low (reference)				
IgG1_GLURP-R2				
High	−2.66	−16.64;5.12	0.626	
Low (reference)				

This table presents the generalized linear model obtained through the control variables bednet use, sex, and HbS. The reference groups corresponded to the low level of IgG1 specific to each merozoite antigen determined according to the median. IgG1 to merozoite *P. falciparum* antigens were quantified at 18 months of age

In bold: significant P value at the 0.05 threshold

**Table 5:** Association between OP and IgG3 to merozoite *P. falciparum* antigens

Opsonic phagocytosis	Coef.	95% CI	P value	
Model 1: OP THP-1				
IgG3_AMA1				Number of observations = 87
High	7.93	−0.38;16.24	0.061	Adj R^2^ = 0.24
Low (reference)				
IgG3_MSP1				
High	−2.68	−9.69; 4.33	0.454	
Low (reference)				
IgG3_MSP3				
High	−0.97	−7.85;5.90	0.781	
Low (reference)				
IgG3_MSP2-3D7				
High	9.54	−1.69;20.76	0.096	
Low (reference)				
IgG3_MSP2-FC27				
High	1.94	−9.63;13.53	0.742	
Low (reference)				
IgG3_GLURP-R0				
High	1.14	−5.74;8.04	0.744	
Low (reference)				
IgG3_GLURP-R2				
High	1.63	−4.71;7.99	0.613	
Low (reference)				
Model 2: OP Neutrophils				
IgG3_AMA1				Number of observations = 87
High	20.4	7.64;33.15	**0.002**	Adj R^2^ = 0.30
Low (reference)				
IgG3_MSP1				
High	−6.12	−16.88;4.63	0.265	
Low (reference)				
IgG3_MSP3				
High	−0.74	−11.29;9.80	0.890	
Low (reference)				
IgG3_MSP2-3D7				
High	7	−10.24;24.25	0.426	
Low (reference)				
IgG3_MSP2-FC27				
High	0.72	−17.04;18.05	0.936	
Low (reference)				
IgG3_GLURP-R0				
High	−5.34	−15.92;5.23	0.322	
Low (reference)				
IgG3_GLURP-R2				
High	5.51	−4.23;15.26	0.268	
Low (reference)				

This table presents the Generalized linear model obtained through the control variables bednet use, sex, and HbS. The reference groups corresponded to the low level of IgG3 specific to each merozoite antigen determined according to the median. IgG1 to merozoite *P. falciparum* antigens were quantified at 18 months of age

In bold: significant P value at the 0.05 threshold

For the subclasses, merozoite-phagocytosis activity was significantly correlated to levels of AMA1 IgG1 (Coef. = 9.38, 95% CI 2.38; 16.39, P = 0.009) and MSP1 IgG1 (Coef. = 7.71, 95% CI 1.81; 13.61, P = 0.010) while levels of AMA1 IgG3 showed a modest association (Coef. = 7.93, 95% CI −0.38; 16.24, P = 0.061). Second, we investigated the association between merozoite-phagocytosis by neutrophils and specific IgG levels (Tables 3, 4, 5). The merozoite-phagocytosis activity was correlated with AMA1 IgG (Coef. = 2.3, 95% CI 0.06;4.54, P = 0.044), AMA1 IgG1 (Coef. = 19.87, 95% CI 9.33;30.40, P = 0.0001), AMA1 IgG3 (Coef. = 20.4, 95% CI 7.64;33.15, P = 0.002), and MSP1 IgG1 (Coef. = 13.21, 95% CI 4.33;22.09, P = 0.004).

### Merozoite-phagocytosis and G3m phenotypes

Since levels of specific IgG3 were associated with merozoite-phagocytosis activity, we determined the distribution of G3m phenotypes in the population (Table 6A). There were a majority of infants carrying G3m5,10,11,13,14 (34.6%), followed by infants carrying G3m5,6,10,11,13,14,24 (27.7%) and G3m5,6,10,11,13,14 (11.9%).

**Table 6:** IgG G3m phenotype distribution (A) and association between OP and IgG G3m phenotypes (B)

A IgG G3m phenotypes	Number (n, %)
G3m5,10,11,13,14	35 (34.6)
G3m5,6,10,11,13,14,24	28 (27.7)
G3m5,6,10,11,13,14	12 (11.9)
G3m5,10,11,13,14,15	3 (3)
G3m5,6,11,24	6 (6)
G3m5,6,10,11,14	6 (6)
G3m5,6,10,11,13,14,15	3 (3)
G3m5,6,10,11,13,15,24	8 (7.9)

B Opsonic phagocytosis	Coef.	95% CI	P value	
Model 1: OP neutrophils				Number of observations = 92
G3m5,10,11,13,14 (reference)				Adj R^2^ = 0.19
G3m5,6,10,11,13,14,24	13.46	2.37;24.54	**0.017**	
G3m5,6,10,11,13,14	−4.31	−18.59;9.96	0.554	
G3m5,10,11,13,14,15	6.86	−17.50;31.22	0.581	
G3m5,6,11,24	2.08	−15.83;19.99	0.820	
G3m5,6,10,11,14	7.82	−13.94;29.58	0.481	
G3m5,6,10,11,13,14,15	29.27	−0.47;59.01	**0.050**	
G3m5,6,10,11,13,15,24	−2.41	−17.58;12.75	0.755	
Model 2: OP THP-1				Number of observations = 92
G3m5,10,11,13,14 (reference)				Adj R^2^ = 0.24
G3m5,6,10,11,13,14,24	7.46	0.31;14.61	**0.041**	
G3m5,6,10,11,13,14	−6.00	−15.2;3.2	0.201	
G3m5,10,11,13,14,15	−0.71	−16.41;14.99	0.929	
G3m5,6,11,24	1.81	−9.73;13.36	0.759	
G3m5,6,10,11,14	2.47	−11.56;16.49	0.730	
G3m5,6,10,11,13,14,15	7.93	−11.24;27.10	0.417	
G3m5,6,10,11,13,15,24	−2.92	−12.70;6.85	0.558	

This table presents the Generalized linear model obtained through the control variables bednet use, sex, birthweight and HbS. The reference group was G3m5,10,11,13,14

In bold: significant P value at the 0.05 threshold

Individuals with the G3m5,6,10,11,13,14,24 phenotype showed higher merozoite-phagocytosis activity by THP1 compared to individuals with the other G3m phenotypes (Generalized linear model, Coef. = 7.46, 95% CI 0.31;14.61, P = 0.041) (Table 5). Children with the G3m5,6,10,11,13,14,24 phenotype (Coef. = 13.46, 95% CI 2.37;24.54, P = 0.017), together with those children with the G3m5,6,10,11,13,14,15 phenotype (Coef. = 29.27, 95% CI −0.47;59.01, P = 0.050), were also associated with high merozoite-phagocytosis activity by neutrophils (Table 6B).

### G3m phenotypes and IgG3 levels against *P. falciparum* merozoites antigens

Infants carrying G3m5,6,10,11,13,14,24 presented higher proportion of infants with a high level of IgG3 against AMA1 and MSP1 (Chi-square test, P = 0.034 and 0.023, respectively). However, a significant low level of IgG3 against MSP2-3D7 was found in infants carrying G3m5,10,11,13,14 (Chi-square test, P = 0.031) (Table 7).

**Table 7:** Association between IgG3 levels to Pf merozoite antigens and IgG G3m phenotypes

	Low level (n, %)	High level (n, %)	P-value ^a^
IgG3 MSP2-3D7			
No G3m5,10,11,13,14	23 (25.3)	40 (43.9)	**0.031**
G3m5,10,11,13,14	17 (18.7)	11 (12.1)	
IgG3 AMA1			
No G3m5,6,10,11,13,14,24	32 (35.2)	34 (37.4)	**0.034**
G3m5,6,10,11,13,14,24	6 (6.6)	19 (20.9)	
IgG3 MSP1			
No G3m5,6,10,11,13,14,24	36 (39.6)	30 (33)	**0.023**
G3m5,6,10,11,13,14,24	7 (7.7)	18 (19.8)	

^a^Statistical significance determined by χ2 analysis

In bold: significant P value at the 0.05 threshold. Only significant P values were shown

## Discussion

The results of the present study showed a higher ability of antibodies from infants included in the CAIG group to stimulate phagocytosis of monocyte cell line THP-1 or primary neutrophils compared to infants belonging to IG and N*Pf*DG groups. OP was previously correlated to malaria protection and judged as a reliable vaccine evaluation criterion through analysis of antibody responses [9, 15, 16, 19, 36], therefore, the observed ability of controlling malaria asymptomatic infection of infants from CAIG group may be related to their high ability to stimulate phagocytosis by OP.

Infants from the CAIG presented higher IgG concentrations against AMA1, MSP1 and GLURP-R2 compared to infants belonging to IG and N*Pf*DG groups (P ≤ 0.001, Additional file 1: Table S1). These results were in line with previous results published on the same cohort in a larger sampling [6]. The higher quantity of opsonizing antibodies targeting merozoites antigens in the CAIG group could explain the better ability to stimulate phagocytosis in vitro. Correlations between high OP values and high concentrations of total IgG against AMA1 and GLURP-R2, IgG1 and IgG3 against AMA1 and IgG1 against MSP1 were observed. In the literature, high levels of IgG1 against AMA1 were independently associated with protection from clinical malaria in Sudan while high IgG3 levels to AMA1 and IgG1 levels to MSP1-19 were found to be strongly predictive of a reduced risk of symptomatic malaria and high-density *P. falciparum* infections in Papua New Guinea [37]. IgG1 to MSP1 was correlated to high OP. Our finding reinforces the work performed by Gonzales et al. [37] and suggest an important role of this subclass in phagocytosis concerning MSP1 vaccine antigen candidate.

The pattern of malaria infections in CAIG group during the survey was very different from the one of IG group. In addition to have the capacity to control their parasitaemia, individuals included in CAIG group presented more asymptomatic infections than IG group (Fig. 2). Infants from CAIG group exhibit higher OP activities at 15 and 18 months of age than infants from IG group. A recent study showed that OP was significantly associated with reduced risk against febrile malaria in Ghanaian children using a prospective design [19]. The higher *in vitro* OP activity observed in CAIG group could reflect the antibody involvement in the control of parasite growth. However, it is difficult to affirm that OP activity observed in this group is responsible for parasitemia control observed during the follow-up. Unfortunately, certain constraints such as the presence of maternal antibodies until 12 month of age and the quantity of available plasma at 15 month of age did not allow us to perform a prospective study.

Immune epidemiological studies have shown that children living in malaria endemic areas may develop naturally acquired immunity against *P. falciparum* malaria after repeated infections. The correlation of high concentrations of IgG subclasses against AMA1 and MSP1 to high levels of OP may reflect the repeated exposure of these children to malaria which can lead to a better antibodies’ memory responses and phagocytosis capacity. Moreover, in this cohort, it has been shown that the levels of IgG against *P. falciparum* merozoite antigens reflected malaria exposure [6]. Thus, this could explain the fact that N*Pf*DG group had lower levels of phagocytosis compared to CAIG and IG groups. Note that a slightly higher bed net possession was observed in N*Pf*DG group, however other factors might be involved in lower exposition to *P. falciparum* infections. However, it is possible that a proportion of infants in N*Pf*DG group could also be protected from malaria infections by other mechanisms.

The finding that the levels of total IgG to GLURP-R2 were associated with a high OP and not significant when considering specific IgG1 or IgG3 may suggest a potential implication of IgG2 and/or IgG4. Indeed, the total IgG level to GLURP was associated with a reduced risk of malaria by Nebie et al*.* [38] or Kana et al*.* [16] while analysis of the IgG sub-classes showed different results.

Indeed, IgG3 and IgG4 against GLURP were associated with a reduced risk of clinical malaria by Nebie et al*.* [38] while only IgG2 and IgG4 against GLURP-R2 were significantly related to a reduced risk of malaria in other studies [39–42]. In a longitudinal cohort study conducted in Ghana, all the GLURP-R0-specific antibodies except IgG2 were associated with an increased OP [16]. IgG2 and IgG4 could potentially be responsible for the effect observed when using purified total IgG. The effect observed with anti-GLURP-R2 total IgG may also be explained by the fact that there was only an effect when IgG sub-classes against GLURP-R2 were combined.

As regards the influence of G3m phenotypes on OP, our results showed a high level of OP associated with the carriages of G3m5,6,10,11,13,14,15 and G3m5,6,10,11,13,14,24. No work previously investigated associations between Gm phenotypes and opsonizing antibodies to *P. falciparum* merozoites. The effector functions of IgG3 are numerous among which activation of complement pathways, neutralization, antigen recognition and cell activation via FcgR receptors [43, 44]. Among IgG subclasses, IgG3 is the most functional, closely followed by IgG1 and this property is due in large part to its strong affinity to FcgR receptors [43, 45]. The efficiency of monomeric IgG3 binding to FcgR on effector cells such as neutrophils and monocytes is the highest. Thus, the interaction of IgG3 with FcgR on these cells leads to a strong immune response in case of infections [45].

Facer [27] was the first author to highlight a relationship between Gm allotypes and malaria by showing a preferential expression of G3m10, G3m11 and G3m14 allotypes associated with a risk of anemia [27]. Migot-Nabias et al*.* demonstrated a protective effect of the Gm5,6,13,14; 1,17 phenotype carriage in Benin [24] while the Gm5,13,14; 3; 23 phenotype (G3m; G1m; G2m) was associated with a high level of IgG1 to *P. vivax Pv*MSP1-19 and *Pv*AMA-1 antigens in Brazilian Amazon endemic area [46]. Moreover, a previous study demonstrated a greater risk of malaria infections when FcgRIIA/FcgRIIIA/FcgRIIIB genotypes were combined to G3m5,6,11,24 and G3m5,6,10,11,13,15,24 phenotypes [26]. G3m5,6,10,11,13,14,24 phenotype associated with high OP may be related to higher IgG3 affinity to FcgR [47, 48] or quantity to AMA1 and MSP1; a significant high level of IgG3 against AMA1 and MSP1 was found in infants carrying G3m5,6,10,11,13,14,24 (Table 7).

This work showed that G3m5,6,10,11,13,14,24 and G3m5,6,10,11,13,14,15 phenotypes were associated with high OP using primary neutrophils while only G3m5,6,10,11,13,14,24 phenotype was associated with high OP in THP-1 cell lines with a decreased significance. Other factors such as FcgR polymorphisms may also impact OP activities. The genotypes of primary neutrophils and THP-1 cells used in this study were respectively FcgRIIA 131 RR/FcgRIIIB NA2NA2 and FcgRIIA 131 RH/FcgRIIIB NA1NA2. It is worth noting that FcgRIIA 131 RR/FcgRIIIB NA2NA2 was previously associated with malaria protection and FcgRIIA 131 RH/FcgRIIIB NA1NA2 with a trend of protection [26].

However, this aspect deserves further studies, performed on diverse populations in high number in order to explore this potential role of Gm phenotypes in the functionality of IgG, which could have a considerable impact in the research of vaccine candidates against malaria.

## Conclusion

This study shows that the group of infants able to control infection had probably a better functionality of IgG through a high OP compared to a control group. It showed also that the inherent polymorphism of the IgG heavy chain represented by the Gm phenotypes play an important role in IgG functionality, opening up avenues for the efficacy of antibodies in the search for anti-malarial vaccines.

## Supplementary Information

**Additional file 1: Table S1. **IgG levels in the study group.

## Data Availability

The datasets used and/or analysed during the current study are available from the corresponding author on reasonable request.
