# Supplementary material for: Naturally acquired antibodies from Beninese infants promote Plasmodium falciparum merozoite-phagocytosis by human blood leukocytes: implications for control of asymptomatic malaria infections
Source: Malar J. 2022 Nov 29;21:356. doi: 10.1186/s12936-022-04361-w (PMC9707106; doi:10.1186/s12936-022-04361-w)
Supplement: Supplementary file 1 — Additional file 1: Table S1. IgG levels in the study group. [file 12936_2022_4361_MOESM1_ESM.docx]

**Supplementary data**

**Supplemental Table 1: IgG levels in the study group**

| IgG (µg/ml) | **CAIG group** | **IG group** | **N*Pf*DG group** | **P value ^a^** |
| --- | --- | --- | --- | --- |
| IgG1_AMA1 | 47.66 (4.66 – 166.47) | 2.94 (0.83 – 10.96) | 0.18 (0.08 – 0.42) | **< 0.001** |
| IgG1_MSP1 | 2.75 (0.76 – 20.19) | 5.86 (0.98 – 25.34) | 0.08 (0.04 – 0.13) | **< 0.001** |
| IgG1_MSP3 | 0.30 (0.86 – 2.91) | 0.29 (0.10 – 1.04) | 0.10 (0.06 – 0.28) | **0.001** |
| IgG1_MSP2-3D7 | 29.41 (2.12 – 53.42) | 3.92 (0.59 – 34.80) | 0.19 (0.11– 0.51) | **< 0.001** |
| IgG1_MSP2-FC27 | 10.14 (3.29 – 59.75) | 2.59 (0.68 – 21.81) | 0.14 (0.08 – 0.22) | **< 0.001** |
| IgG1_GLURP-R0 | 1.07 (0.48 – 7.66) | 0.62 (0.31 – 1.72) | 0.30 (0.17– 0.89) | **0.026** |
| IgG1_ GLURP-R2 | 2.36 (0.43 – 10.94) | 0.46 (0.17 – 2.19) | 0.14 (0.06 – 0.44) | **0.002** |
|  |  |  |  |  |
| IgG3_AMA1 | 0.23 (0.08 – 1.46) | 0.08 (0.01 – 0.29) | 0.01 (0.009 – 0.03) | **< 0.001** |
| IgG3_MSP1 | 0.08 (0.01 – 0.31) | 0.07 (0.01 – 0.48) | 0.007 (0.001 – 0.02) | **0.001** |
| IgG3_MSP3 | 0.03 (0.01 – 0.17) | 0.01 (0.003 – 0.06) | 0.008 (0.002 – 0.04) | **0.043** |
| IgG3_MSP2-3D7 | 1.54 (0.10 – 2.94) | 0.14 (0.03 – 2.07) | - 1. (0.003 – 0.02) | **< 0.001** |
| IgG3_MSP2-FC27 | 1.45 (0.22 – 2.79) | 0.10 (0.03 – 2.01) | 0.01 (0.003 – 0.02) | **< 0.001** |
| IgG3_GLURP-R0 | 0.02 (0.002– 0.05) | 0.01 (0.004– 0.04) | 0.005 (0.003– 0.02) | 0.597 |
| IgG3_GLURP-R2 | 0.10 (0.01 – 0.29) | 0.01 (0.006 – 0.06) | 0.007 (0.0007 – 0.01) | **0.001** |

^a^ Statistical significance determined by Kruskal-Wallis test

In bold: significant P value at the 0.05 threshold.
